# Decoding of spatiotemporal activity of auditory information in the cortex

**DOI:** 10.1186/1471-2202-12-S1-P139

**Published:** 2011-07-18

**Authors:** Yusuke Hara, Yoshiki Kashimori

**Affiliations:** 1 Graduate School of Information Systems, University of Electro-Communications, Chofu, Tokyo 182-8585 Japan; 2 Dept. of Engineering Science, University of Electro-Communication, Chofu Tokyo 182-8585 Japan

## 

Animals utilize auditory information for survival and communications of conspecifics. A sequence of sound is analyzed in animals’ brain as elementary components such as notes and syllables. It has been reported that auditory information is represented by spatiotemporal activity of primary auditory cortex [1]. However, how the elementary components of sound are encoded from the spatiotemporal activity of neurons is poorly understood. To address this issue, we present a model of auditory cortex, which performs a hierarchical processing of auditory information. The model consists of three layers of 2-dimensional networks. We show that the aspects of the spatiotemporal activity in the primary cortex are encoded by a combination of feature-detective neurons and then by a dynamical attractor in higher-order cortex. The present study provides a clue for understanding the mechanism of how the information of notes and syllables are constructed from spatiotemporal activity of the primary auditory cortex.

We propose a neural network model for hierarchical processing of auditory information, which consists of three networks. The auditory information is encoded with spatiotemporal pattern of neuronal activity in the primary auditory (A1) cortex. The model of A1was made based on the previous model by Taniguch and Yamaguchi [2]. Then the spatiotemporal aspect of the neuronal activity is detected by the feature-detective (FD) neurons in the second layer. These FD neurons integrate the spatiotemporal pattern over a short time period, thereby enabling the second layer to represent the information about notes and about the correlation of notes. The information encoded by the FD neurons is then combined as a linkage of attractors in the feature binding (FB) layer, providing a semantic information such as word.

## Results

The model of the primary auditory cortex reproduced well the spatiotemporal activity of A1 neurons. The FD neurons integrated the spatiotemporal activity over a short time period and encoded the information about notes (i.e. “a”,”b”, “c”) and the correlation between the notes. The FB network combined the information about notes and their correlations as a linkage of dynamical attractors, enabling the network to represent the two words, “a-b-c” and “c-b-a”. After the perception of ‘a-b-c’ or ‘c-b-a’, the network state recovered to a background state, in which the network state exhibited an itinerant state between the attractor “a-b-c” and “c-b-a”.

## Conclusion

We have shown a network model for hierarchical information processing in the auditory cortex, which consists of three layers. These layers encoded the information about different aspects of auditory features along the pathway from A1 to FB layer, enabling the system to percept a word in the FB layer. The present study provides an insight to understanding the information processing in auditory cortex.
